# Is hypoalbuminemia a risk factor for small bowel anastomotic leaks in infants? A multivariate analysis

**DOI:** 10.1007/s00383-026-06539-8

**Published:** 2026-07-13

**Authors:** Stefanie Langreen, Marie Uecker, Sebastian Juhl, Jens Dingemann, Rim Kiblawi

**Affiliations:** 1 Department of Pediatric and Adolescent Surgery, Hannover Medical School, Hannover, Germany Carl-Neuberg-Straße 1, 30625; 2 SAP SE, Walldorf, Germany; 3 Truman School of Government and Public Affairs, University of Missouri, Columbia, MO USA

**Keywords:** Anastomotic leak, Small bowel anastomosis, Hypoalbuminemia, Anastomotic integrity, Fluid management

## Abstract

**Purpose:**

Anastomotic leaks (AL) are critical complications following bowel anastomosis with an incidence of up to 10.5% in infants. Previous studies in animal models and adults have suggested a link between postoperative fluid overload and hypoalbuminemia with anastomotic leaks. In infants, the role of hypoalbuminemia on anastomotic integrity after small bowel anastomosis remains unclear.

**Methods:**

Retrospective study of infants < 1 year, undergoing elective small-bowel anastomosis between 2015 and 2024 (Ethics No.-11362-_BO_K_202). Data on demographics, weight gain, serum albumin levels and use of diuretic medication during the first five postoperative days were collected. Univariate and multivariate analysis were performed to detect risk factors for ALs in infants.

**Results:**

13 of 219 patients (5.9%) developed ALs. Demographics were similar comparing patients with and without leaks. In univariate analysis, serum albumin levels were significantly lower (20.3 g/l vs. 26,3 g/l, *p* < 0.0001) in patients with AL compared to patients without AL and multivariate analysis confirmed a significant association between low serum albumin levels and AL occurrence,

**Conclusion:**

In infants, small bowel anastomotic leaks are associated with low postoperative serum albumin. Whether hypoalbuminemia should be regarded as an early warning sign of an undiagnosed, developing anastomotic leak or represents a causal risk factor can only be determined with further studies.

**Supplementary Information:**

The online version contains supplementary material available at 10.1007/s00383-026-06539-8.

## Introduction

 Small bowel anastomosis (SBA) with end-to-side (E/S) or end-to-end (E/E) anastomosis may be required in newborns and infants for various reasons, with the most common indications being necrotizing enterocolitis, spontaneous intestinal perforation, different forms of intestinal atresia [1–4], but also during reconstructive surgery.

While SBA is generally considered a safe procedure, anastomotic leaks (AL) remain a critical complication, particularly in infants, with rates ranging up to 10.5% [5–8]. If left untreated, AL potentially leads to severe complications, such as sepsis and death [5].

The successful healing of gastrointestinal anastomosis depends on various factors, such as tension-free approximation of the bowel ends and maintenance of adequate blood supply to the tissue [9]. While for hand sewn bowel anastomoses operative techniques, surgeons experience and length of surgery are crucial and well known aspects to consider, other patient characteristics need to be taken into account [10] : Newborns with low birth weight are more likely to develop postoperative complications [11], in particular anastomotic leaks [10]. Poor nutritional status (patients with congenital heart disease for example, are often affected due to increased metabolic demand paired with reduced energy intake and genetic factors [12]) is also known to play a role in the development of postoperative complications including AL [13–17].

Notably, postoperative fluid overload seems to play a crucial role in AL as it has been linked to it in adults and in animal models, possibly by diluting necessary coagulation factors and immune cells, as well as by increasing postoperative edema in the anastomotic site, hence limiting perfusion [18, 19]. Furthermore, a drop in serum albumin level has been identified as poor prognostic marker for the outcome of elective intestinal surgery in animal and adults [20]. 

Albumin is an essential protein in maintaining the oncotic pressure, substance transport and has antioxidant and anti-inflammatory values, substantial to wound healing of intestinal anastomosis [21–24]. In neonates and infants however, permissive hypoalbuminemia is commonly practiced by tolerating lower serum albumin levels to avoid possible side effects, such as anaphylactic reactions, hypervolemia and impaired oxygen exchange [25–28]. 

To date no study in infants has been performed to investigate the role of serum albumin levels on anastomotic integrity. We hypothesized that hypoalbuminemia is a predictor of AL after SBA in infants and aimed to determine a clinically significant cut-off level for postoperative care. In order to establish a reliable value, we included only patients after elective surgery without underlying sepsis or critical illness.

## Materials and methods

A retrospective case-control study of infants under 1 year of age, undergoing elective hand-sewn small-bowel anastomosis, E/E anastomosis for ileostomy closure or E/S anastomosis (as part of Kasai portoenterostomy) in our department of Pediatric Surgery was conducted from January 2015 until July 2024. Ethical approval (No.-11362-_BO_K_202) was obtained. All patients’ files were reviewed through the hospital informatics system.

Exclusion criteria were patients aged ≥ 1 year, those undergoing any type of bowel anastomosis other than SBA, patients receiving small bowel anastomosis for intestinal atresia or during emergency laparotomy, those undergoing revisional surgery and patients with sepsis or heart failure.

Small bowel anastomoses were performed with absorbable sutures (braided polyglactin 4 − 0 or 5 − 0), in a single-layer manner for both, E/E and E/S anastomosis, running suture or interrupted stitches were used according to surgeons’ preference.

Due to the patients’ young age, all infants were briefly admitted to our pediatric or neonatal intensive care unit for postoperative monitoring and transferred to the regular pediatric surgical ward within 24 h. Weight changes and urinary output were routinely monitored and documented in the patients’ charts during the first 5 postoperative days. The postoperative weight was compared preoperative weight and calculated in percentage of the body weight.

For patients after E/S anastomosis following Kasai-portoenterostomy an additional standardized routine protocol was followed, including a five-day course of antibiotics, usually Cefotaxime, and scheduled blood samples for albumin, liver and renal functions, and inflammatory markers (CRP, IL-6 and complete blood count). Patients with E/E anastomosis for ileostomy closure were administrated a three-day antibiotic therapy, (Ampicillin-Sulbactam), and blood samples were collected in patients with decline in clinical conditions. Oliguria (< 1 ml/kg/h) or excessive weight gain (> 20%) would prompt the administration of diuretics in all patients, serum albumin levels < 30 g/L would routinely lead to i.v. substitution with human albumin (20%) at 1 g/kg, as per standard practice on the pediatric surgical ward.

The primary endpoint was the correlation of postoperative serum albumin levels with anastomotic leaks after SBA in infants, and the identification of a clinically relevant cut-off albumin serum level. Serum albumin levels measured between postoperative day three to five were included in this analysis. For patients who received albumin, only values obtained before first substitution were considered. In patients with AL, only values obtained before diagnosis of AL were included. AL was defined as a defect at the anastomotic site resulting in leakage of bowel content and revisional surgery. Secondary endpoints included the association of other factors as postoperative weight gain within the first 5 days, diuretic administration, body weight, Z-score corrected for gestational age (GA) and cardiac defects. Postoperative weight was compared with preoperative weight and expressed as a percentage change relative to the preoperative body weight. For term-born patients weight for age Z-scores were calculated using the WHO growth standards [29]. In preterm patients, weight-for age Z-scores were corrected for postmenstrual age and calculation was based on the INTERGROWTH-21st Preterm Postnatal Growth Standards [30]. Statistical analysis was performed using the open-access software R. Quantitative variables are represented as mean ± standard deviation (SD) unless stated otherwise and assessed by a Welch t-test. Qualitative variables were assessed using the Chi-Square Test. Multivariate analysis was performed using Firth’s penalized logistic regression model [31, 32]. P-values < 0.05 were considered significant.

## Results

### Univariate analysis

#### Patient demographics and perioperative data

In total, 219 patients were included in this data analysis. 126 received small intestinal E/S anastomosis and 93 patients received elective E/E anastomosis. 30% of our collective were born under 37 weeks gestational age. Overall, 13 patients developed small bowel anastomotic leak (5.9%). Mean age at surgery was 80.9 days ± 58.3, mean weight was 4093 g ± 1466.5 and gestational age - corrected Z-score was − 1.56 ± 2.2. Age at surgery, weight and Z-score did not differ between those with or without anastomotic leaks. For cardiac defects without heart failure and for anastomosis type (E/E vs. E/S), one-sample proportions test with continuity correction indicated no statistically significant difference between the groups.

In addition to the comparable anastomotic leak rates between E/E and E/S anastomoses, postoperative serum albumin levels were similar between these two procedures as well. In contrast, E/S anastomosis in patients with Kasai portoenterostomy experienced a significantly greater weight gain (16.8 ± 7.8 vs. 7.8 ± 6.4, < 0.001*) and a higher need for diuretic administration (55% vs. 14%, < 0.001*).

**Table 1:** Patient demographics, gestational age (GA), one-sample proportions test with continuity correction for cardiac defects

	No anastomotic leak *n* = 206	Anastomotic leak *n* = 13		*p*-value
Age at surgery (d)	80.4 ± 57.7	89.4 days ± 69.4		0.658
Weight at surgery (g)	4057 ± 1486.6	4644.6 ± 1424		0.068
GA-corrected Z-score for weight	-1.6 ± 2.3	-1.6 ± 0.90		0.853
Associated Cardiac defects %	12.6	7.7		0.852
Proportion of E/E anastomosis among groups (%)	56.3%	76.9%		0.242

* if *p* < 0.05

#### Postoperative data

In univariate analysis, a significant difference in serum albumin levels and CRP levels was identified between patients without AL compared to the group of patients that had developed AL. Differences in weight gain as well as diuretic administration were not significant at our chosen confidence level of *p* < 0.05.

**Table 2:** Postoperative data

	No anastomotic leak *n* = 206	Anastomotic leak *n* = 13	*p*-Value
Serum Albumin (g/L)	26.3 ± 4.7	20.3 ± 3.6	< 0.001**
Postoperative weight gain (% of body weight)	12.9 ± 8	19.9 ± 13	0.091
CRP (mg/L)	47.9 ± 41.5	165.2 ± 63.4	< 0.001**
Diuretic medication (%)	41.3	69.2	0.095

* if *p* < 0.05, ** if *p* < 0.01

When comparing patients by surgical technique, no significant difference in leak rates between patients after E/E and E/S anastomosis was detected, despite the latter patients (patients with Kasai portoenterostomy) demonstrating significantly higher weight gain and more frequent diuretic use. In univariate analysis, no significant difference in serum albumin levels was found between the two groups.

**Table 3:** Postoperative Data End-to-end (E/E) vs. End-to-side (E/S) anastomosis

	E/E anastomosis *n* = 93	E/S anastomosis *n* = 126	*p*-Value
Anastomotic leak (%)	3.2	7.9	0.242
Serum Albumin (g/L)	26.7 ± 5.7	25.5 ± 4.7	0.344
Postoperative weight gain (% of body weight)	7.8 ± 6.4	16.8 ± 7.8	< 0.0001**
Diuretic medication %	16.7	61.4	< 0.0001**
Associated cardiac defect %	14	12.7	0.941

* if *p* < 0.05, ** if *p* < 0.01

### Multivariate analysis

The multivariate regression analysis was conducted to model the AL presence as a function of the covariates discussed here. Since the outcome is binary and 5.9% of patients developed AL, we estimated a Firth’s logistic regression model that provides unbiased coefficient and marginal effect estimates even in small samples. Parameter estimates are obtained using penalized maximum likelihood [31, 32]. After removing observations with incomplete data, 100 cases remained in the analysis.

Results revealed that, low serum albumin levels were significantly associated with a higher risk for anastomotic leaks.

**Table 4:** Firth’s penalized Logistic regression model (risk factors)

	Estimate	Standard error	*p*-value
(Intercept)	10.07	3.8	0.001**
Postoperative % Weight gain	0.04	0.04	0.349
Serum albumin level	-0.51	0.15	< 0.001**
Diuretic administration	1.77	1.09	0.062
E/S anastomosis	-2.84	1.36	0.027*
Underweight (Z-Score <-2)	0.88	1.1	0.420
Cardiac defects	-1.35	1.66	0.426

*if *p* < 0.05, ** if *p* < 0.01

Table 5 in the appendix demonstrates results from an alternative model specification, showing that the effect of serum albumin is robust to the inclusion of CRP in the model. Additionally, the appendix contains an additional sensitivity analysis which demonstrates the unlikeliness of an omitted confounder invalidating the main result.

Furthermore, we report the results from the analysis using multiple imputation instead of listwise deletion in the appendix (Table 6). The key findings remain robust. Additionally, the fraction of missing information was examined to evaluate the impact of missing data on precision. Missing information had a minor impact (< 0.10) on almost all parameter estimates. Only the coefficients associated with diuretic medication (0.13) and E/E anastomosis (0.20) have moderate information losses due to missingness. Therefore, these parameter estimates should be interpreted with caution.

In Table 7 in the Appendix, we share the results of the regression model for E/E and E/S separately, demonstrating that the results of the main analysis are generally applicable.

To simplify the interpretation of our results from a clinical perspective, we used the estimates from our main regression model and conducted a simulation to demonstrate the impact of serum level albumin on the risk of AL development in patients. Specifically, we calculated the predicted probability of an AL for a range of different albumin levels, while holding the other variables constant. In the simulated scenario, we specified a case with weight gain of 16%, which is the mean gain in our patient cohort, diuretic medication, E/S anastomosis, not underweight at surgery, and no associated cardiac defects. We then varied the serum albumin level from 14 g/L to 32 g/L to see how changing this factor influences the predicted leak risk. Using the coefficient estimates as well as the estimates variance-covariance matrix from the model, we sampled 1000 sets of simulated coefficients for each albumin level from a multivariate normal distribution to accurately account for sampling uncertainty.

**Fig. 1:**
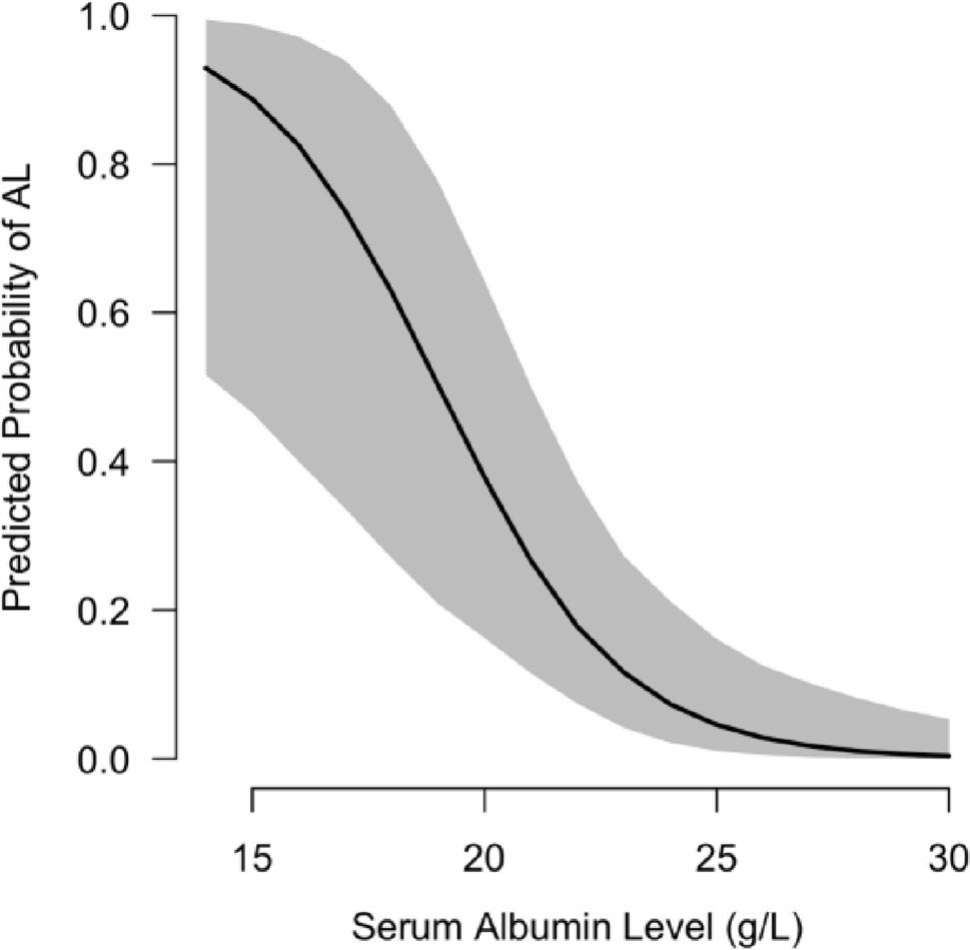
Predicted probability of AL at different levels of serum albumin. Other variables are set to: 16% weight gain, use of diuretics, E/S anastomosis, no underweight, no associated cardiac defects. Shaded area indicates the simulated 95% confidence interval based on 1000 simulation iterations for each scenario

The result clearly demonstrates that higher serum albumin levels are associated with a marked reduction in the risk of anastomotic leaks. Based on our regression model, the predicted probability of anastomotic leaks in patients with serum albumin level of or above 30 g/L approaches 0 (Fig. 1).

## Discussion

Despite the heterogenous nature of indications for small bowel anastomosis in neonates and infants, obscuring the identification of consistent risk factors for anastomotic leaks, we demonstrated a strong association between postoperative hypoalbuminemia and the development of AL in infants.

In our analysis, hypoalbuminemia was identified as predictor for AL. This supports the idea that fluid retention and weight gain may be a consequence of low serum albumin rather than an independent risk factor. Similar interpretations have been proposed in other neonatal studies, where hypoalbuminemia preceded clinical signs of sepsis or fluid imbalance [33, 34]. 

The role of hypoalbuminemia as a potential risk factor for AL is well-documented, as it contributes to disbalance in oncotic pressure and fluid homeostasis consequently leading to edema of the bowel wall and impaired wound healing [21, 24, 35]. Nonetheless, no specific albumin threshold associated with AL has been identified for infants so far. In patients older than 3 years, hypoalbuminemia is typically defined as a serum albumin level below 30 g/L [28], while in neonates, serum albumin levels vary with gestational age [36]: For full-term neonates (gestational age above 37 weeks), serum albumin levels above 25 g/l are considered normal [36]. However, particularly in preterm neonates (up to 29.2% of NICU admitted patients demonstrate serum albumin levels ≤ 25 g/l [37]), evidence from randomized controlled trials is limited and does not support the routine administration of albumin to reduce mortality or morbidity associated with hypoalbuminemia [25]. 

Thus, albumin supplementation is avoided if possible [38]. While serum albumin levels around 25 g/L may be acceptable in otherwise healthy neonates, their impact on anastomotic healing remains unexplored.

Higher serum albumin levels are associated with less AL in infants after SBA: Our model predicts substantially lower leak risk at albumin levels ≥ 30 g/L. These results are consistent with previous findings in in animal models and adults [13, 20, 25, 26]. 

As part a negative acute-phase protein, decreased serum albumin occurs simultaneously with increased C-reactive-Protein (CRP) or leukocytosis as a response to inflammation, this makes establishment of a clear causal relationship difficult [39]. Given the inverse correlation between CRP and albumin [34], one might hypothesize that hypoalbuminemia reflects underlying sepsis or inflammation rather than acting as an independent risk factor [35]. However, while CRP serum levels were not included in our main model, additional analyses presented in the Appendix (Table 5) show that incorporating CRP values at different time points does not affect the association between low serum albumin levels and the development of AL.

Nevertheless, the retrospective nature of our study prevents us to establish a causal relationship between hypoalbuminemia and anastomotic leakage. To minimize reverse causation, only serum albumin levels obtained before the diagnosis of anastomotic leakage and before albumin substitution were included. Whether patients with low serum albumin levels had already developed a subclinical, undetected anastomotic leak at the time of sampling cannot be ruled out. Therefore, postoperative hypoalbuminemia may represent a predisposing risk factor for anastomotic leakage or and early laboratory finding in cases of present AL. Regardless, our findings suggest that postoperative presence of hypoalbuminemia should warrant clinical evaluation for early signs of AL.

Despite anastomotic leak rates appearing higher in patients undergoing E/S anastomosis during Kasai-portoenterostomy, the difference was not statistically significant in both univariate and multivariate analysis. This patient group demonstrated significantly greater postoperative weight gain and higher rates of diuretic use, due to fluid retention, as they typically suffer from hepatopathy and chronic hypoalbuminemia [40, 41]. Serum albumin was identified as an independent risk factor for anastomotic leaks in our multivariate analysis. Indeed, contrary to our expectations, patients undergoing E/S anastomosis showed a trend towards fewer leaks in logistic regression analysis, holding constant other factors as hypoalbuminemia and weight gain. However, this observation must be interpreted with caution. Patients in our cohort undergoing E/S anastomosis, typically those with biliary atresia, received more standardized early follow-up, including more aggressive albumin monitoring and substitution. In contrast, patients undergoing E/E anastomosis had a less regulated regimen for blood testing or albumin supplementation.

This in turn should emphasize the importance of standardized perioperative protocols. They help reduce variability in patient care, improve overall outcomes and reduce length of hospital stay [42–45]. Implementing a standardized perioperative protocol across the different units could potentially improve patient safety and quality of care [43]. Including routine blood samples with focus on serum albumin levels for all neonates and infants undergoing small bowel anastomosis may help mitigate the risk for anastomotic leaks. Such a protocol could be beneficial not only for patients with hepatopathy (e.g. in patients with biliary atresia) but also for other infants with predisposition to hypoalbuminemia, who may not receive an extensive biochemical monitoring. Even though this strategy may demand significant resources and coordination among multidisciplinary teams [46], we believe that if designed carefully, the benefits may outweigh possible disadvantages.

In order to validate such albumin substitution, prospective studies would be needed, however we believe that our results justify its careful consideration. A strict postoperative protocol could regulate indication, dosage and method of albumin administration. The risks of possible allergic reactions could be mitigated by providing a standardized operating procedure and proper training [26]. By adhering to a strict intravenous liquid substitution protocol, the risk of albumin induced fluid overload and (pulmonary) edema would be anticipated early on and decreased [38]. 

The retrospective nature of our data analysis allows for variability in the follow-up protocols. Additional potential biases, such as differences in surgeons experience and surgical technique, cannot be entirely excluded. It is also important to note that E/E anastomoses are considered a teaching procedure at our institution, typically performed by junior surgeons under the supervision of a consultant, whereas an experienced, dedicated surgical team generally performs E/S anastomosis as part of Kasai Portoenterostomy. Furthermore, we included both patients with E/E and E/S anastomoses in our analysis, and the differences in these anastomotic techniques could have influenced the outcomes. In our cohort, patients were managed by different units during the first postoperative days, including the PICU, NICU, and pediatric surgical ward, each with its own fluid and nutritional protocols. This likely contributed to inconsistent albumin monitoring and supplementation. Potential confounding factors, such as underlying pathology and surgical indications must also be considered when interpreting the results.

We were able to collect a relatively large sample size within a single-center setting, which helps minimize bias related to surgical techniques and ensures a more uniform approach to postoperative care. Further prospective studies with standardized perioperative protocols and systematic data collection are needed to provide stronger evidence regarding the risk factors for anastomotic leaks in neonates and infants.

## Conclusions

Low postoperative serum albumin levels in infants and newborns after small bowel anastomosis are associated with anastomotic leaks.

Randomized controlled studies are necessary to determine whether, consistent perioperative monitoring of serum albumin and fluid management in patients undergoing intestinal anastomosis could help identify or even reduce the incidence of AL in infants.

## Supplementary Information

Below is the link to the electronic supplementary material.

Supplementary Material 1

## Data Availability

No datasets were generated or analysed during the current study.
