# Supplementary material for: Is hypoalbuminemia a risk factor for small bowel anastomotic leaks in infants? A multivariate analysis
Source: Pediatr Surg Int. 2026 Jul 13;42(1):301. doi: 10.1007/s00383-026-06539-8 (PMC13364815; doi:10.1007/s00383-026-06539-8)
Supplement: Supplementary file 1 — Supplementary Material 1 [file 383_2026_6539_MOESM1_ESM.pdf]

8 APPENDIX AND SUPPLEMENTARY MATERIALS

Regression Models

As outlined in the main text, it is possible that e.g., low serum albumin may be a symptom of ongoing inflammation not an independent factor. However, due to the relatively small sample size (n = 100) and concerns regarding multicollinearity given the high correlation between serum albumin levels and the inflammatory marker C-reactive-Protein (CRP) with a pearson correlation coefficient of -0.41 in our sample, we excluded CRP values from the main model. To validate the robustness of our findings to this alternative causal explanation, we estimate an additional regression model which, in addition to all variables in the main model, includes the maximum CRP value in the first six days after the surgery. The following table reports the results.

	Estimate	Standard Error	p-value
(Intercept)	1.79	4.02	0.561
Postoperative % Weight gain	0.06	0.05	0.215
Serum albumin level	-0.29	0.16	0.029**
Diuretic administration	0.74	1.26	0.550
E/S anastomosis	-1.16	1.39	0.404
Underweight (Z-Score <-2)	0.73	1.35	0.612
CRP (mg/L)	0.02	0.01	0.004***
Cardiac defects	-0.64	1.76	0.690

***Table 5**: Firths penalized logistic regression model (risk factors),* * if p<0.05, ** if p<0.01.

While CRP has, as expected, a statistically significant positive effect on the development of AL, the effect of serum albumin remains statistically significant, although the effect strength decreases.

Sensitivity Analysis

The relatively small sample size and high levels of multicollinearity limit the ability to add a number of additional control variables to the model which introduces the possibility of an omitted variable bias. In short, if a variable that is correlated with both, the development of AL and serum albumin levels is omitted from the model, the correlation between these two variables may be spurious which would invalidate our findings.

In order to assess the likelihood of omitted variables bias in our model, we performed a sensitivity analysis. The aim was to describe the properties an omitted variable has to have in order to render the impact of serum albumin on the development of AL insignificant.

Since our treatment, serum albumin levels, is continuous, we use a linear regression model instead of the penalized logistic regression model in the main text. The linear model includes all the variables in the main model. Serum albumin is statistically significant (coefficient estimate = -0.03; p-value = 0.00015) with a partial R-squared of 0.144. In our sensitivity analysis, we are looking for an omitted variable that reduces the effect estimate of serum albumin level. We find that an unobserved confounder variable would have to explain at least 18.35% of the residual variance of serum albumin levels and the outcome to be strong enough to render the effect of serum albumin levels insignificant at a confidence level of 95%. A confounder that explains at least 33.46% of the residual variance would be needed in order to bring the point estimate of serum albumin to 0.

Given the partial R^2 of serum albumin with the development of AL is 0.144, we regard it as unlikely that any unobserved confounder reaches these thresholds. Therefore, we conclude that our main result is insensitive to the omission of important confounders.

Multiple Imputation

To address missing data and assess the robustness of our findings, we conducted a sensitivity analysis using multiple imputation by chained equations (MICE). Missing data were imputed for the following variables: percentage weight gain, serum albumin levels, the use of diuretic medication, E/S anastomosis, underweight status, and cardiac defects.

We created five imputed datasets (m = 5) with ten iterations to ensure convergence. The imputation model included all variables from the primary analysis reported in the main article. Logistic regression models with Firth's bias-reduction method were fitted separately to each imputed dataset. Parameter estimates and standard errors were pooled using Rubin's rules to obtain final inference.

Results from the multiple imputation analysis are presented in Table 6. The pooled estimates were consistent with those from the primary analysis, with similar directions and comparable magnitudes. The fraction of missing information ranged from 3% to 20% across parameters, indicating that missingness had minimal to moderate impact on inference. The highest share of missing information was observed for E/S anastomosis (20%). Overall, these findings support the robustness of our primary results and suggest that conclusions are not substantially affected by the missing data mechanism.

	Estimate	Standard Error	p-value	Missing Information
(Intercept)	6.55	2.63	0.013*	0.08
Postoperative % Weight gain	0.04	0.04	0.336	0.05
Serum albumin level	-0.45	0.12	0.000**	0.06
Diuretic administration	0.86	0.86	0.320	0.13
E/S anastomosis	-0.18	0.99	0.853	0.20
Underweight (Z-Score <-2)	0.70	0.85	0.411	0.09
Cardiac defects	-0.91	1.34	0.500	0.03

Table 6*: Firths penalized logistic regression model (risk factors) using multiple imputation. Five imputed datasets created using multiple imputation by chained equations. Estimates pooled using Rubin's rules.* * if $p<0.05$, ** if $p<0.01$.

Separating E/E anastomosis and E/S anastomosis

Since E/E anastomosis and E/S anastomosis patients constitute quite different populations with distinct biological conditions, we ran the same regression model with listwise deletion as in our main analysis for the two populations separately. This allows the effect of the other predictors to vary between the two types of patients. The following table compares the coefficient estimates from the two samples.

	E/E anastomosis (n = 13)	E/S anastomosis (n = 87)
(Intercept)	-0.71	6.83*
Postoperative % Weight gain	0.17	0.04
Serum albumin level	-0.16	-0.46**
Diuretic administration	1.86	1.12
Underweight (Z-Score <-2)	1.53	0.11
Cardiac defects	-0.75	-0.19

***Table** 7: Firths penalized logistic regression models (risk factors) for patients with E/E anastomosis and E/S anastomosis.* * if $p<0.05$, ** if $p<0.01$.

While none of the estimates from the model for patients with E/E anastomosis are statistically significant, assessing the significance level (and also the magnitude of the coefficient estimates) is misleading since it is based on only 13 observations. More importantly, the direction of the estimates is consistent between the two models. This indicates that, while the strength of the effects may differ between the populations, the results of the main analysis are generally applicable.
